# “ . . . I Should Maintain a Healthy Life Now and Not Just Live as I Please . . . ”: Men’s Health and Fatherhood in Rural South Africa

**DOI:** 10.1177/1557988315586440

**Published:** 2016-07-08

**Authors:** Victoria Hosegood, Linda Richter, Lynda Clarke

**Affiliations:** 1 University of Southampton, Southampton, UK; 2 Africa Centre/University of KwaZulu-Natal, KwaZulu-Natal, South Africa; 3 DST-NRF Centre of Excellence in Human Development, University of the Witwatersrand, South Africa; 4 Human Sciences Research Council, Durban, South Africa; 5 Department of Population Health, London School of Hygiene and Tropical Medicine, London, UK

**Keywords:** men, health, fathers, qualitative, South Africa

## Abstract

This study examines the social context of men’s health and health behaviors in rural KwaZulu-Natal, South Africa, particularly in relationship to fathering and fatherhood. Individual interviews and focus groups were conducted with 51 Zulu-speaking men. Three themes related to men’s health emerged from the analysis of transcripts: (a) the interweaving of health status and health behaviors in descriptions of “good” and “bad” fathers, (b) the dominance of positive accounts of health and health status in men’s own accounts, and (c) fathers’ narratives of transformations and positive reinforcement in health behaviors. The study reveals the pervasiveness of an ideal of healthy fathers, one in which the health of men has practical and symbolic importance not only for men themselves but also for others in the family and community. The study also suggests that men hold in esteem fathers who manage to be involved with their biological children who are not coresident or who are playing a fathering role for nonbiological children (social fathers). In South Africa, men’s health interventions have predominantly focused on issues related to HIV and sexual health. The new insights obtained from the perspective of men indicate that there is likely to be a positive response to health interventions that incorporate acknowledgment of, and support for, men’s aspirations and lived experiences of social and biological fatherhood. Furthermore, the findings indicate the value of data on men’s involvement in families for men’s health research in sub-Saharan Africa.

## Introduction

There is increasing recognition that understanding men’s health and health behaviors requires close consideration of the social context (Hirsch, 2007; Lohan, 2007; Treadwell et al., 2012). Considerable research attention has focused on the influence of masculinity in shaping men’s health, for example, to understand how men construct and enact health and health behaviors (see, e.g., Courtenay, 2000; O’Brien, 2005). However, fewer studies have explicitly addressed the interrelationships between men’s health and men’s experiences of family life, fathering, and fatherhood (Palkovitz, 2002; Robertson, 2007; Williams, 2007, 2010). Research and practice exploring the intersections between men’s health, gender, and the family environment can contribute to effective public health strategies to promote the health of families, as well as the health of young men (Connell, 2012; Engle, 1997; Robertson & Williams, 2010). The aim of this study was to explore Zulu-speaking men’s own accounts of fathering and fatherhood in order to understand whether family involvement influences men’s own health and health behaviors. The authors draw on transcripts of interviews and focus groups (FGs) conducted with Zulu-speaking men from a rural area of KwaZulu-Natal, South Africa.

Evidence from observational studies in the United Kingdom and the United States point to a generally positive effect of fatherhood on men’s health (Bartlett, 2004; Garfield, Clark-Kauffman, & Davis, 2006; Henwood & Procter, 2003; Levine & Pitt, 1995; Palkovitz, 2002). Analyses of data from longitudinal family studies have identified positive impacts of fatherhood on happiness, feelings of contentment, and self-esteem (Knoester, Petts, & Eggebeen, 2007; Umberson & Gove, 1996). Fatherhood can influence men’s health through reductions in risk-taking behaviors (smoking, alcohol) and promote positive behaviors (diet, exercise; Umberson, 1987, 1989). However, negative effects of fatherhood have also been reported, for example, anxiety and depression among fathers associated with new responsibilities and relationship conflict (Booth & Amato, 1991; Gove, 1973; McLanahan & Adams, 1987, 1989; Ramchandani, Stein, Evans, & O’Connor, 2005; Reichman, Corman, & Noonan, 2004). Through analyses of fathers’ accounts of health experiences in the United Kingdom and the United States, researchers have described the considerable influence on men’s health and health practices of fulfilling and combining work and fathering roles. Particularly, when men’s abilities or health are compromised, these can present health challenges, for example, through elevated stress, tiredness, and exhaustion (Watson, 2000; Williams, 2007).

Qualitative studies in the United Kingdom report that men themselves tend to emphasize the “good” or “positive” influence that becoming a father has on men’s health and health behaviors (Robertson & Williams, 2010). Distinctive patterns of behavior have been identified in men’s narratives about their health and health behaviors related to the periods before and after becoming a father (Robertson, 2006, 2007; Williams, 2007). The period before fatherhood is often described as a carefree time in which excessive drinking and hard partying is common and acceptable (referred to as *release*). In contrast, the period after becoming a father is described as a time when men should and often do behave more sensibly and moderately (referred to in terms of *control*). Robertson (2007) has identified two types of drivers in men’s health behaviors associated with fatherhood. The first are changes that are “rule-based,” that is, seen as necessary to become a good, dutiful, partner and father. These are often related to high-risk behaviors, especially drinking and smoking. The second set of changes are driven by a specific desired health outcomes, for example, when a father wants to be healthy so that he can play with his children or live to see them grow up.

### Men and Families in KwaZulu-Natal

In South Africa, residential separation of Black fathers from their children is commonplace (Morrell, 2006). In northern KwaZulu-Natal, population-based studies conducted in the past decade report that approximately two thirds of all resident children do not belong to the same household as their fathers at birth (Hosegood, McGrath, Bland, et al., 2009). Similar findings have been reported in studies using national household survey data (Posel & Devey, 2006; Richter et al., 2010). Both colonial and *apartheid* political, economic, and social policies entrenched circular labor migration and strongly racially segregated urban settlements. During the *apartheid* era, the families of labor migrants were largely restricted to rural *Bantustans* or “homelands” under the authority of traditional authorities (Spiegel, 1996; Townsend, Madhavan, & Garey, 2006). Marriage rates in KwaZulu-Natal are among the lowest in sub-Saharan Africa, with high rates of extramarital fertility (Hosegood, McGrath, & Moultrie, 2009; Hunter, 2006; Preston-Whyte, 1978).

In South Africa public and media discourses, Black fathers in dispersed family arrangements are generally assumed to have little or no involvement with their children (Prinsloo, 2006). This assumption reinforces stereotypes of contemporary, Black men as irresponsible partners and fathers who neglect family responsibilities (Prinsloo, 2006; Richter, Corman, & Noonan, 2010; Swartz & Bhana, 2009). In contrast, quantitative and qualitative studies in South Africa have reported that men, even those who are not resident with their children, are often positively engaged in financial support, care work, children’s education, and family health under diverse family circumstances (Desmond & Hosegood, 2011; Montgomery, Hosegood, Busza, & Timaeus, 2006; Morrell & Jewkes, 2011; Madhavan, Richter, Norris, & Hosegood, 2014). A high degree of contact and support has also been observed between nonresident fathers and children (Madhavan & Townsend, 2007; Madhavan, Townsend, & Garey, 2008). Furthermore, scholars have emphasized the profound and persistent importance of fatherhood in the construction of African, and more specifically Zulu, adult male identity (Hunter, 2006, 2008; Lesejane, 2006; Lindegger, 2006; Mkhize, 2004, 2006; Morrell, 2006). Given the high incidence of orphaning during childhood due to parental deaths from AIDS and other causes, as well as repartnering, many men will also take on the role of father for nonbiological children (Hosegood & Madhavan, 2010; Mkhize, 2004, 2006; Richter et al., 2011). In father scholarship, the term “social father” has been used to refer to men who consider themselves to be involved as fathers with nonbiological children (Engle & Breaux, 1998). Social fathers are described by Morrell (2006, p. 14):

Such men might be in situations of formal adoption, or in a living relationship with the mother of the children, or a member of an extended family who has taken on the role and responsibilities of caring for children (for example, a man’s brother might see himself as having the responsibility of father when his male sibling is out of work, or because he is the older son).

Unlike terms such as stepfather, adopted father, and foster father, “social father” is an inclusive term that does not define the man’s relationship to the child with respect to the child’s mother or a legal status (Hosegood & Madhavan, 2010).

South African men have one of the highest rates of early mortality <60 years in the world with a combination of severe health threats, most notably HIV, tuberculosis, and injuries (Garrib, Herbst, Hosegood, & Newell, 2011; Rajaratnam et al., 2011). In contrast to the extensive research conducted in South Africa, focusing on men’s sexual and health-related behaviors as risk factors for adverse health outcomes in female partners and children (Hosegood & Desmond, 2011; Richter, 2010), scant attention has been paid to understanding the relevance of men’s relationships, identities, and involvement in families shaping their own health and behaviors (Desmond & Hosegood, 2011; Hosegood & Madhavan, 2010; Morrell & Richter, 2006).

## Method

Data collection was conducted in two phases between 2008 and 2010. The first phase of the study explored the social context and community perceptions of fatherhood, identity, father involvement with children and families, and the impact of fatherhood on men’s health and well-being. A single in-depth community informant interview was conducted with 13 men who were Zulu-speaking, were living in the Umkanyakude district, and were knowledgeable about local communities. Community informants were recruited following introductions by community liaison staff, members of the research organization’s local community advisory board, and by follow-on introductions from study participants. Community informants were aged between 30 and 70 years and were diverse in social, economic, and faith characteristics. Most community informants were active in local tribal authority governance or voluntary leaders of other community organizations. Using a similar recruitment strategy, an additional 28 Zulu-speaking men living in the district were invited to participate in three FGs that were arranged on the basis of age and parenting status: a group of 10 older men, 40 to 60 years; a group of 6 men, 30 to 40 years, all of whom were active in community groups, most men were fathers and half were unemployed; and a group of 12 younger men <30 years who were fathers. The majority of older men participating in the FGs described themselves as fathers.

The community informant interviews and FGs were structured by separate topic guides that covered themes including the attributes and roles of fathers, Zulu traditions related to fathering and fatherhood, roles and responsibilities of nonresident fathers, media and public representations of men and fathers, and men’s health and health behaviors across the life course. The purpose of conducting FG discussions after interviews with community informants was to explore the extent to which knowledge and beliefs regarding men’s roles in families were also shared by a diverse group of men who were not identified for any particular tribal or community position.

In the second phase of the study, two in-depth interviews were conducted with 10 men (25-46 years) who were biological fathers of at least one primary school–aged child (typically not more than 12 years of age) and who reported themselves to be involved as fathers in the lives of one or more biological or nonbiological children (see Table 1). The sample of fathers were diverse with regard to family arrangements, employment status, education, marital status, coresidence with children, relationship status with the mother(s) of their biological children, and whether acting as social fathers to other children (see Table 1). Four fathers were included who were members of households in the Umkanyakude district, but were living in Durban at the time of the study. The first and second in-depth interviews with fathers were loosely structured by separate interview topic guides. The topic guides used in the second phase were developed building on the findings of the first phase community informant interviews and FG discussions. The topic guides were finalized and translated in an iterative process that included team discussion, consultation with the community advisory board, and other research colleagues, as well as piloting draft topic guides with volunteer respondents. Given the wide variety of possible family circumstances in which participants could be living, the authors needed to balance flexibility in the structure of the topic guides with a degree of consistency in wording. Thus, broad questions were included, as well as more detailed questions should a specific line of inquiry open up.

**Table 1.** Description of Participants in Individual Father Interviews.

Father #	Age (years)	Children^a^	Other characteristics
F1	40	6 Children; with Mother 1 (14 years, 16 years), with Mother 2 (14 years), with Mother 3 and wife (2 years, 4 years); coresident with these children; social father to niece (30 years) and her two children (infants)	Employed, married
F2	33	4 Children; with Mother 1 (10 years, 4 years), with Mother 2 (3 years), with Mother 3 (1 year); not coresident with children of Mother 1, coresident with 3 years and 1 year children; in a relationship with Mother 3; social father to coresident niece and nephew older adolescents	Employed, not married
F3	46	3 Children (16 years, 19 years, 20 years); coresident with all children and their mother; social father to 2 nephews (28 years, 8 years) and a niece (10 years); also, coresident with these children	Temporary work, married
F4	39	2 Children (10 years, 3 years); coresident with children; social father to 1 niece (16 years) who is coresident with him	Employed, married
F5	25	1 Child (6 years); not coresident with child nor in a relationship with child’s mother	Casual employment, not married
F6	36	2 Children (7 years, 10 years); coresident with children and their mother	Self-employed not working, married
F7	33	1 Child (6 years); in a relationship with child’s mother, not coresident with child	Student, married
F8	31	1 Child (12 years); not coresident with child nor in a relationship with child’s mother; social father to 2 nieces (16 years and 17 years) who live elsewhere	Self-employed, not married
F9	34	2 Children; with Mother 1 (8 years), with Mother 2 (2 years); not coresident with children	Employed, not married
F10	33	1 Child (5 years); not coresident with child nor in a relationship with child’s mother	Employed, not married

a Children refers to each man’s biological children unless noted.

The first interview with the father’s childhood parenting experiences charted his current relationships with families and children, residential arrangements, levels and types of father involvement with biological and nonbiological children, relationships and engagement with child’s mother, coparent, caregivers, kin and in-laws. In the first interview, general questions were asked in order to gather spontaneously reported data about the types of activities or behaviors men considered to constitute father involvement. A life history map was generated during this interview to represent the connections between, and timing of, union formation and dissolution, fathering and family arrangements, migration, education, and employment. During the second interview conducted with seven of the fathers, emerging themes from the man’s first interview were revisited and more specific questions were asked to gather data about the types of roles and activities in relation to children and other family members. These included activities performed by men during weekdays, weekends, and holidays; with children of different ages, gender, coresidence, and family histories; activities done together with the child inside (chatting, watching TV, working together, praying) and outside the household (attending church or school, visiting people, shopping, driving). Men were asked about whether they performed tasks such as bathing, feeding, and dressing children often as part of day-to-day tasks or under particular circumstances. Questions were also asked about activities that can be performed from a distance (calling to speak to, or about, the child with someone else, thinking or speaking about a child, praying for them) or together with other people (having a *braai* [barbeque], visiting relatives, playing sports). In the second interview, additional topics included men’s health and the impact of family life on men’s own health.

All community informant interviews and FGs were conducted in meeting rooms in research offices in either Umkanyakude individual interviews, whereas some interviews with fathers were conducted in a research office in Durban. All participants received a small reimbursement to compensate for travel and other incidentals. Five people with higher education-level training in social sciences conducted the interviews and FGs over the course of the project—three men and two women aged between 25 and 41 years. Written informed consent was given by all participants with separate permission sought to record and transcribe the interviews or FG. All FG discussions and most interviews were conducted in Zulu; a few interviews were conducted in English at the request of the participants.

All interviews and FG discussions were taped and transcribed verbatim. Transcripts in Zulu were translated into English by the study team and professional translators. Data coding was systematic and thematic, based on an iterative process of coding material for explicit topics prioritized a priori to meet specific study objectives, but also coded themes that emerged during the interviews or process of coding and reflection (Ritchie & Spencer, 1994). The coding was performed by the first author who also wrote summaries of the interview material for each father participating in the in-depth interviews, as well as analytical notes and commentaries. These documents were discussed with the second author. Later stages of analysis involved cross-referencing codes and rereading material to recheck data against analytical interpretations (Crabtree & Miller, 1999). NVivo 9 software QSR was used to manage data, coding, analytical notes, participant summaries, and commentaries.

## Results

Three main themes related to the topic of the interrelationships between fathering, fatherhood, and men’s health were identified through analyses of transcripts: (a) the interweaving of health status and health behaviors in descriptions of “good” and “bad” fathers, (b) the extent of positive presentations of men’s own health during interviews, and (c) positive transformative narratives and perceptions around health in men’s accounts of fatherhood. This article draws on findings from analyses of all interview and FG transcripts from the first and second phases, concentrating particularly on the accounts of fathers during individual interviews when discussing Themes b and c. In this article, unless specified, the term “father” or “fatherhood” refers to biological or social fathers. All names are pseudonyms.

### Health Status and Health Behaviors in Descriptions of “Good” and “Bad” Fathers

All respondents associated “healthy” behaviors (moderation, self-control, being proactive) with characteristics of “respected” fathers (responsible, balanced, and self-respecting). Many respondents emphasized concepts related to respect when describing fathers’ health behaviors. A central theme was that men are only able to fully fulfil their roles and responsibilities as fathers when they are respected by families and community. A widely reported view among respondents was that this respect can only be achieved if men show a high degree of self-respect and respect for partners and children. Showing self-respect and respect for others necessarily required men to be in good health and not to behave in ways thought to threaten men’s own health and that of their children and partners.

The two dominant, juxtaposing characterizations of widely described by community informants and individual fathers were those of the “good” and “bad” father. Health status and health behaviors were frequently discussed when differentiating fathers on the basis of these identities. “Good” fathers were perceived to be “healthy” men, not only with regard to their own health status but also by behaving in ways that did not affect negatively on their children and partners or, even better, that positively promoted family health. This image of good fathers—health-conscious themselves and actively engaged in ensuring their family’s health—was contrasted with “bad” fathers whose health status and behavior posed a threat to the health and well-being of their partners and children. Poor health and health behaviors, particular those associated with degeneracy or immorality (notably heavy drinking and sexual promiscuity), were also described as revealing deficiencies in a man’s character that would undermine his success and respect as a father.

My personal opinion on a father who does not look after his family is that he must be seriously sick at least mentally. Honestly! How on earth can a good father in good health refuse to look after his family? Any way what can I say, it happens that you find that a father is working but his wife and children are out there looking for food from neighbours. This is not acceptable. [ . . . ] A father should be a person who behaves well in front of the community who does not involve himself in negative things, who is an example to his children and the community in general. (Male, community informant, 70 years, #CI11)

In contrast, the characterizations of “bad” fathers provided in individual interviews and FG discussions were very colorful and detailed (“hairless animals”; “itchy men”). While respondents spoke of other fathers, in some cases even their own fathers, in such terms, no fathers applied these terms to themselves. Two behaviors, in particular excessive alcohol use and multiple sexual partners, were frequently used to illustrate irresponsible behaviors by fathers. Many respondents emphasized drunkenness and sexual promiscuity as threats to men’s own health and that of their families but also as practices that undermined men’s ability to be “good” fathers by causing them to lose respect from their families, communities, and, importantly, their own self-respect.

Excessive or hazardous alcohol use was a theme associated with negative descriptions of fathers in all individual and FG discussions. Respondents related excessive drinking to a wide range of characteristics including unpredictability, violence, self-absorption, unreliability, shambolic appearance, coarseness and harshness, choosing to spend time with drinking friends rather than with his family, financial costs, and risks to employment (“Something that I see from others [ . . . ] with whom I work, a man passes by where he lives, perhaps to go and drink in the afternoon. Err . . . he doesn’t even know what happens to his child during the day”). Being a moral father was held to be an integral part of a being a respected father. An older FG participant spoke in terms that were echoed by many other men:

Let us put this into perspective, if we say that, “Mr. Gumede is a good father,” then he mustn’t come home drunk every time because whenever he’s speaking, no one really listens to him. He would also smell of beer to his children and they would say “He smells and how can we accept such a person as a father, he is drunk—its better when we see him in the morning.” We would always say that this is [ . . . ] a bad father.” (Male, 40-60 years, #FG2 participant)

The strong terms in which alcohol use was described by some respondents was often linked to a clear moral position or personal experience. In discussing their own childhood, several men described negative experiences due to fathers who drank heavily, men who were violent or neglectful and of whom they were scared.

Other behaviors frequently evoked in FGs and interviews to demonstrate control, self-respect, or respect for a partner were those related to sexual behavior. Married men, in particular, emphasized faithfulness; however, many other ways were cited of showing women respect such as listening and understanding their opinions (“ . . . a father need to be someone who respect opinions of other people regardless of gender”). These statements by a father of three older children referred to several pertinent themes—the need for a father to respect himself and his family and the inappropriateness of uncontrolled behavior:

. . . the first thing, as a father is to respect yourself within the household. A father cannot be found to be all over the place, like a father who likes a lot of women. A father should hold himself together. [ . . . ] The role of a father as I have said [ . . . ] is to support my family with everything. To respect them too. I am not a father that stirs up [negative] emotions. I don’t fight with my family, I don’t know how to fight with my family. I am the kind of father who is polite; I also see that they follow my lead.

Sex is what I see as a problem nowadays, I view sex as a problem. It now has a negative impact. For a father to be involved in multiple relationships, to have random sex that is a problem. If they can look at it as I do, perhaps these problems can be reduced. (Father, 46 years, #F3)

Many participants described their own experiences with, or recognition of, another type of Zulu father—“old time Zulu fathers.” Although such fathers were often described as having strong personal morals and to have been respected; the dominant theme was about the “fear” that these men inspired in their children, wives, and other people.

So, what I learnt from my father is that you have to work hard if you are a man, so that your family can enjoy being alive. He did spend time with us but we didn’t enjoy being with him. [ . . . ] Even though he was an approachable man he used to hit us a lot. *Eigh* my father, I could say that he was honest because no matter how small the issues he would want to know that how much you contributed to it. A child that lies *err* . . . he did not like it. (Father, 39 years, #F4)

Frequently used descriptions of the behavior of Zulu fathers in the past included their “distance” from children and the family, not thinking it necessary to talk with or understand their children, or using harsh punishments without hesitation. With a few exceptions, the archetypal severe Zulu father of the past was not held up by participants as a positive role model.

[Previously] fathers would not have accepted closeness between the father and the child. We too were scared, you would fear him even if he was not saying a word, you just fear being close to him; this is unless you were helping him with something and he just wanted to send you to get him whatever he wanted. (Father, 31 years, #F8)

[ . . . ] my father was a feared father. So even when there was something important I wanted to say, that was troubling me, or I wanted to ask him I would not say it. [I worried] what would he do? If I tell him it might cause problems. I don’t want my children to go through the same thing—for them to be afraid to tell me something no matter how minor it is. (Father, 39 years, #F4)

All men interviewed were affiliated to one of the Western- or African-initiated Christian congregations common in the local area, including the Zulu Nazareth Baptist Church or Shembe Church. For some men, religious beliefs were important in shaping their perception that smoking, drinking, and particular types of sexual behavior are immoral. Several respondents reported that they did not drink alcohol (“teetotal”) because of their religious beliefs, for example, Seventh Day Adventists, or because of their own experiences growing up of the negative effects of alcohol on men and their families.

### Men’s Accounts of Their Own Health

When talking about health, men considered a broad range of issues, encompassing aspects of physical health (disease, disability, nutritional status) and psychological health or well-being (stress, anxiety, emotional insecurity, a positive outlook). In the individual interviews, all fathers emphasized the excellent status of their health. “I feel strong and healthy,” “I don’t have any illnesses,” and “I’m not a person that gets sick regularly” were characteristic of men’s descriptions of themselves. Evidence of “good health” was provided in terms of men’s health status (fit, strong, never ill) but also by descriptions of his behavior which he considered to be positive for men’s health. The health behaviors most frequently mentioned by men were watching one’s diet, taking exercise, health checkups, and abstaining from, or engaging in, moderate drinking and smoking; that is, types of behaviors that demonstrated moderation were restraint and willpower, being proactive by screening for health problems, or following a strong personal moral code of conduct. When men combined descriptions of positive health behaviors with accounts about their involvement in the family, there was a sense that these particular men, and any others who behaved like them, were able and well-suited to fulfilling their family roles and responsibilities (“Wherever I am, I can jump and go”; “I’m a safety conscious person most of the time. Even on the road my habits are different from other people. When I predict that something might be dangerous and avoid it”).

None of the fathers reported chronic health problems and many stressed how little, if at all, they made use of health services (“I’m not used to hospitals. I’ve been there only once and only because I got hurt playing soccer”). In the few cases in which men described interacting with health service providers, the focus was not on the nature of the illness but rather on the respondents’ proactive role in keeping himself healthy. When describing health problems that had occurred in the past, fathers stressed their determination and resolve in controlling or eliminating the problem (“I cured myself,” “I willed myself to get well”). While no men described interacting with doctors or nurses (e.g., consultations or counseling and testing), many men spoke positively about medical technology that screened, measured, and checked their health, some with apparent misunderstandings:

I also check my BP [blood pressure] from time to time. I do it at the clinics, just to see the results of my health. It is something that I do just to see whether there is any difference compared to last time. This helps to maintain a decent lifestyle. (Father, 46 years, #F3)

No . . . I’m healthy, I’m very healthy. As I have said when I was talking about my children; I try to instill a healthy life in them. I take good care of my health most of the times, as to how I eat. I also do what is called ECG, checking the whole body system, including HIV and AIDS, asthma, what-what-what, TB and all those things. I am not a passive person, and say that [the sickness] will eventually fade away. I need to find out what is making me sick. I don’t want to do guess work, so that I can know if what is making me cough is TB or if is something else then I need to take care of it rapidly. (Father, 36 years, #F6)

No respondent discussed HIV in anything like the openness with which they shared their views and spoke about their own behaviors related to diet, weight, smoking, and alcohol. No participant disclosed his own HIV status nor that of any other family member.

### The Impact of Fatherhood on Men’s Health

Some men spontaneously discussed the transformative effect of becoming a father on their lifestyle generally, and more specifically on their health, in the first interview. This theme was raised with all respondents during the second interview. Fathers described their desire and actions to change their health practices on becoming a father: “I think it is important to reduce alcohol consumption, use of drugs and reducing the number of sexual partners [on becoming a father]”; “What I learnt is that I should maintain a healthy life now and not just live as I please because I am in a different stage”; “I think I learnt my lesson because now I maintain a proper lifestyle. The way that I live now is much better. I am not promiscuous with my health life.” This man involved as a father to his biological son and two of his brothers’ children described the changes he made after his son’s birth.

I can say that it started when I became a father—to take care of what I eat. I used to eat without care but I have learnt to maintain a good life by changing how I eat. I have a belief that a person can live to whatever age they wish. That is if he changes the way that he eats and behaves. That has become part of my life—to be respectful, respect life, and to maintain such things. I think that my goal and focus changed after this realization. I can say that I can see a difference comparing myself and what I do now at this stage of fatherhood. (Father, 31 years, #F8)

The necessity for fathers to put their “old ways” behind them to perform well as a father was strongly supported by community respondents. A young man in an FG said:

Let me put it this way, if a father hasn’t changed his old ways, there isn’t much that he can accomplish as a father. Everyone would want to see someone doing positive things in life—tomorrow your child will indicate that my father did something special in his life so commend his behavior and attitude. Older people sometimes put pressure on young fathers to change their behavior. (Male, <30 years, #FG3 participant)

However, a few fathers appeared to reject any sense that their health behaviors had been different in the past. Their narratives emphasized, and often appeared to overemphasize, a lifelong commitment to a healthy lifestyle. For these fathers, the lack of, or moderate impact of, fatherhood on their health mirrored men’s accounts of having grown and matured through their experiences as fathers but from a solid starting point rather than after a wild or intemperate youth:

Respondent: “I didn’t drink [before]. Even before I used to try and take care of what I eat and not just eat whatever.”

Interviewer: “So there is no impact or influence?”

Respondent: “No. What I wish I could do is more exercise, as it is something that I’m not doing to par, I think the rest of it is going well.” (Father, 39 years, #F4)

Although there were no reports of major physical or mental ill-health as a consequence of becoming a father, many men spoke about increased worries, anxiety, and pressure. Fathers not only spoke about feeling stressed but that their ability to handle stress was particularly important in their ability to perform their family roles well.

Being a father it’s demanding. It’s demanding because there are a lot of things to focus on. The first thing that I do when I wake in the morning is to take care of my children. How do I do it? So all of that, when I’m not financially stable I can’t do everything properly. I need to have money or there should be money around so that I could be in a right state of mind. Not that I am sometimes crazy, but it’s just that when things don’t go my way I get agitated. I become short tempered, because I don’t have money; not to have money is a problem. What puts pressure on me is that sometimes there is something that I need to sort out using money. [ . . . ] If there is something that is hanging like a cloud, which puts pressure [on me]. (Father, 36 years, #F6)

During interviews in Zulu, when fathers spoke about issues related to stress, they frequently used English psychological terms:

Fathers want to have a place where they meet and just talk and free their minds because most of them have been affected by stress. [ . . . ] Even if they can just sit and plan that today we will talk about the nature of the family problems and unpack problems that happen at home. [ . . . ] This one and that one, discuss how can we solve them [ . . . ] and be trained. (Male, community informant, 30 years, #CI3)

Fathers also spoke about how being actively involved in promoting and protecting the health of children positively benefited their own emotional and physical health. Fathers expressed pride at being actively engaged in promoting their children’s health and well-being (“A father definitely feels great when he has children who are alive and healthy”). This was particularly marked for those fathers who saw their roles as going beyond that of providing food or access to health care to that of having a positive influence through inspiration, being a role model, providing guidance, and as an educator. Fathers’ interest in their children’s diet and exercise can benefit men and their children as this account illustrates:

Something that I talk about a lot that I think is a priority to me, its health issues. “What is it that you are eating? Is it healthy? Is it junk?” I encourage my children that they must drink a lot of water. You must eat veggies; you must eat fruits. That is what I always try to focus on, to make them watch out. They must be clean, they must be neat, their hair mustn’t be untidy. [ . . . ] So, part of being health conscious is that I tend to talk about a lot with them. Sometimes with a child there are things that a parent will see they need. It is like exercising, my son doesn’t like it. [ . . . ] But he has gotten used to it, he enjoys it. We do exercises and sit ups, push-ups too and all of those things. Even when we haven’t gone to the sports field and are just sitting on the sofa, I will ask him to put his feet under the sofa and say “Please do twenty sit-ups, please do twenty push-ups.” (Father, 36 years, #F6)

Despite more than half of the fathers not being coresident with at least one of their children, respondents appeared to prefer to talk about the positive aspects for their health of being engaged positively with children with whom they were resident rather than the more challenging experiences related to children living elsewhere. A few fathers, for whom day-to-day oversight and intervention in the health of their children was constrained or not possible, referred to the stress and anxiety caused by their lack of immediate knowledge about or perceived inability to protect their children from afar.

## Discussion

This research is one of only a few studies to focus directly on the interrelationships between men’s health and their identity and involvement as fathers in sub-Saharan Africa. Zulu-speaking men identified multiple positive impacts on men’s health and behaviors of fatherhood. The narratives were very similar to those reported in research with men in the United Kingdom and the United States, for example, reductions in alcohol consumption, eating better, staying home in the evening, and having fewer sexual partners (Palkovitz, 2002; Robertson, 2007; Robertson & Williams, 2010). In the resource-poor setting of rural KwaZulu-Natal, fatherhood also affected men negatively through increased stress, anxiety, and worry. Stress was often related to efforts to meet personal and social expectations, financially support, and about wanting but feeling unable to protect their children and families adequately. Being in a healthy emotional and physical state was advantageous in surviving and overcoming these stresses. In one of the few commentaries on the subject in a public health journal, Garfield et al. (2006) also noted the complex and dynamic benefits and pressures of fatherhood on men’s health.

### Respectful and Respected

Being respected is central to men’s ability to be a “good” father. Participants had highly consistent idealized father identity that include morally and culturally appropriate behaviors, including health-related behaviors. These attributes are essential if men are to gain the highly desired respect of one’s children, family, and community. The accounts resembled the concept of “respected identity” as documented elsewhere and which is needed for individuals to develop self-respect and self-esteem (Simon, 2004). The way in which discourses about morality and “good citizenship” as fathers shapes men’s concept of health or enactment of health practices has been reported elsewhere (Robertson & Williams, 2010). Drawing on the findings from UK studies of diverse groups of men, including fathers, Robertson and Williams (2010) note:

Lifestyle risk discourses carry with them moral connotations and messages about the appropriate and required (health) behaviours expected of “good citizens” (Lupton, 1993). These are found in men’s empirical accounts in terms of what people “ought to do”, what they know they “should do”, or indeed in terms of presenting themselves as virtuous citizens who do “the right thing”, or do not do the “wrong” thing (p. 55).

Being healthy was considered to be of considerable practical and symbolic significance for fathers and their families. In contrast, being in poor health or behaving in ways that increased men’s own or their families susceptibility to disease or injuries were frequently discussed as threats to achieving or maintaining respect; most prominently behaviors related to risky sexual behavior or heavy drinking. Although not specifically focused on fathers, an ethnographic study of men working in a mining town in rural Eastern Uganda similarly identified that men were concerned that their ability to perform masculine social roles would be threatened losing the respect of their family and society should it come to be known that they had taken an HIV test or treatment (Siu, Seeley, & Wight, 2013).

### Zulu Father Identities

In writing about African dimensions of fatherhood, Mkhize (2006) has described fatherhood as

. . . an identity project immersed in social, cultural, historical and economic contexts. Fatherhood is intertwined with the process by means of which men come to an understanding of who they are—their sense of identity and place—in society (p. 186).

Most participants asserted that they themselves understood and behaved in a very different way to Zulu fathers in the past. Many told stories about their own fathers to illustrate the kind of father that they did not want to be. Zulu fathers of earlier generations were described by participants very similarly with attention drawn to them having been absent, disinterested in their children’s lives, distant, disciplinarian, violent, or self-interested—fathers who received respect only by demanding it from their children and families rather than earning it through their actions and behavior. Participants, particularly younger men, placed considerable emphasis on their aspirations to be fathers valued for being engaged, caring, communicative, responsible, and respectful. These findings support those from two other recent South African studies of men’s involvement in families in highlighting the development of a contemporary, alternative masculinity among African men, which is being shaped by an active assumption of an identity of *men who care* for their children and families (Makusha, Richter, Knight, Van Rooyen, & Deevia, 2013; Morrell & Jewkes, 2011). The consensus among participants that their generation’s cultural norms and fathering styles are markedly different to those of men in earlier generations resembles research on social change in the United States during the 1980s and 1990s (see, e.g., Coltrane & Parke, 1998; LaRossa, 1988).

For the Zulu men, the context of the severe HIV epidemic in KwaZulu-Natal may be salient in the extent to which participants placed emphasis on link between respect and health behaviors, notably sexual activity and alcohol. Research in South Africa has highlighted the way in which public, policy, and research statements about men as the drivers of the HIV epidemic, the impact of the epidemic being less for men than for women and children, or that men play little or no role in mitigating the consequences of HIV in affected families, go largely unchallenged (Hosegood & Desmond, 2011; Richter, 2010). These findings are also comparable with the “responsible citizen” identity identified in narratives of Zimbabwean men accessing HIV treatment by Skovdal et al. (2011) in which participants presented themselves as taking good care of both their own health and their families.

### Strengths and Limitations

The current study has several limitations. Observational data were not collected on men’s involvement in families, health status, or health behaviors. The extent to which men’s reported and actual health status or health behaviors align could not be examined. Thus, it is important to reflect on whether bias may have been introduced through the way men represented themselves and their experiences in the research setting generally, and specifically in relation to the study topic. On enrolling in the study, men frequently mentioned being pleased that researchers were finally speaking to fathers and not just mothers. Almost all participants approached the interviews and FG discussions with considerable seriousness and gravitas. All fathers in this study reported a large amount of involvement with biological and nonbiological children, even when not living with them or being in a relationship with the child’s mother. Men with little interest in fatherhood, or who had experiences about which they were unhappy or ashamed, are very likely to be underrepresented in this study. Men were aware that the study would be looking at issues related to fathers’ and men’s experiences of fatherhood, and while no men refused to participate during the informed-consent process, those with little interest or for whom the topic was emotionally difficult may not have come forward to participate.

Similarly, few men in this study described their current health and health behaviors as anything other than exemplary. In contrast, in a study of young (17-24 years), Black and colored fathers from two different communities elsewhere in South Africa, researchers reported that men openly discussed engaging in unprotected sex, drinking, and drug use (Swartz & Bhana, 2009). The limited number of accounts that included descriptions of irresponsible or uncontrolled behavior before fatherhood also do not mirror those of studies in the United Kingdom using lay narratives from men about the health over the life course (see, e.g., Williams, 2007, 2010). The participants in the current study were from a wide age range and were older and more established in their work and/or family lives than the young fathers reported by Swartz and Bhana (2010). Many were employed, most had more than one child, many had responsibilities for nonbiological children, some were married, and several were considered to be the head of their own households. Men worked hard during discussions and interviews to distance themselves from negative images of “African men” or “African fathers” that they themselves presented collectively and individually. Their accounts of these “other” men typically included reference to heavy drinking, promiscuous, unhealthy, HIV-infected, uncaring, and violent. Although all participants were living in or strongly connected to an area with a very high prevalence of HIV, men spoke much more often about weight and smoking as important risks to their health during the individual interviews. However, HIV and AIDS were frequently mentioned during the community informant interviews and FG discussions suggesting that men are more comfortable discussing HIV as a general, rather than personal, health risk.

The current findings point to four areas where future work on health promotion and research on men and families is needed. First, the findings justify the project aims and study objectives by identifying that fathering and fatherhood is strongly related to men’s perceptions and desire for improving their own health and that of their children and other family members. The current study provides evidence supporting calls for increased efforts in sub-Saharan Africa to engage men, particularly fathers, in family-based health interventions and programs (Desmond & Hosegood, 2011; Engle, 1997; Richter et al., 2009; Sherr, 2010). Second, the experiences when approaching and engaging men and women around this research topic suggest that interventions that explicitly acknowledge men’s aspirations and experiences of social and biological fatherhood would be very positively received in these communities. Third, drawing similar conclusions to those suggested by qualitative studies on fathers and health in other countries (Richardson, 2010; Robertson, 2003), this study suggests that health literacy and promotion interventions targeting men in South Africa should explore how best to incorporate the widely held view of interconnections between a man’s health, health behaviors, and a respected identity as a man and father. Lesejane (2006) notes that the traditional image of fatherhood in southern Africa offers a potentially “restorative” model for redefining fatherhood and improving the relationship between men, mothers, children, and other members of their families. However, there is a risk that men deviating from these ideals of “good” and “healthy” may feel challenged or excluded from health and family support initiatives. Fourth, this study provides further evidence that nonresident Black fathers in South Africa have and desire positive involvement with children. This underscores the value of collecting data about nonresident fathers in household-based surveys and studies in communities where dispersed families are common (Bennett, Hosegood, Newell, & McGrath, 2015; Hosegood & Madhavan, 2010).

In concluding, researchers and health providers are encouraged to avoid stereotyping South African Black fathers as disengaged family members uncaring about their own health. The current study suggests that evaluation research is warranted to test whether the effectiveness of interventions to promote men’s health can be improved by designs and content that acknowledge and support men in their diverse and often complex family roles and circumstances.
